# The shared biomarkers and pathways of systemic lupus erythematosus and metabolic syndrome analyzed by bioinformatics combining machine learning algorithm and single-cell sequencing analysis

**DOI:** 10.3389/fimmu.2022.1015882

**Published:** 2022-10-19

**Authors:** Yingyu Wang, Zhongzhou Huang, Yu Xiao, Weiguo Wan, Xue Yang

**Affiliations:** ^1^ Division of Rheumatology, Huashan Hospital, Fudan University, Shanghai, China; ^2^ Institute of Rheumatology, Immunology and Allergy, Fudan University, Shanghai, China

**Keywords:** systemic lupus erythematosus, metabolic syndrome, bioinformatics, machine learning, hub genes, single-cell

## Abstract

**Background:**

Systemic lupus erythematosus (SLE) is one of the most prevalent systemic autoimmune diseases, and metabolic syndrome (MetS) is the most common metabolic disorder that contains hypertension, dyslipidemia, and obesity. Despite clinical evidence suggested potential associations between SLE and MetS, the underlying pathogenesis is yet unclear.

**Methods:**

The microarray data sets of SLE and MetS were obtained from the Gene Expression Omnibus (GEO) database. To identify the shared genes between SLE and MetS, the Differentially Expressed Genes (DEGs) analysis and the weighted gene co-expression network analysis (WGCNA) were conducted. Then, the GO and KEGG analyses were performed, and the protein-protein interaction (PPI) network was constructed. Next, Random Forest and LASSO algorithms were used to screen shared hub genes, and a diagnostic model was built using the machine learning technique XG-Boost. Subsequently, CIBERSORT and GSVA were used to estimate the correlation between shared hub genes and immune infiltration as well as metabolic pathways. Finally, the significant hub genes were verified using single-cell RNA sequencing (scRNA-seq) data.

**Results:**

Using limma and WGCNA, we identified 153 shared feature genes, which were enriched in immune- and metabolic-related pathways. Further, 20 shared hub genes were screened and successfully used to build a prognostic model. Those shared hub genes were associated with immunological and metabolic processes in peripheral blood. The scRNA-seq results verified that *TNFSF13B* and *OAS1*, possessing the highest diagnostic efficacy, were mainly expressed by monocytes. Additionally, they showed positive correlations with the pathways for the metabolism of xenobiotics and cholesterol, both of which were proven to be active in this comorbidity, and shown to be concentrated in monocytes.

**Conclusion:**

This study identified shared hub genes and constructed an effective diagnostic model in SLE and MetS. *TNFSF13B* and *OAS1* had a positive correlation with cholesterol and xenobiotic metabolism. Both of these two biomarkers and metabolic pathways were potentially linked to monocytes, which provides novel insights into the pathogenesis and combined therapy of SLE comorbidity with MetS.

## Introduction

Systemic lupus erythematosus (SLE) is one of the systemic autoimmune diseases characterized by a loss of tolerance and excessive autoimmune reaction, with an increasing number of atypical, early, or comorbid cases (1). The typical clinical symptoms of SLE include red speckles on the skin and multiple organ involvement, mostly in young women (2). However, the etiology of SLE, which may be related to genetic predisposition, environmental exposure, gender, or some endogenous triggers, is exceedingly complicated and has not been uncovered thoroughly (3). Previous research has been implicated that some genes as the biomarker candidates of SLE, including IFI27, CXorf21, NCF1-339, et al. (4–6). Metabolic syndrome (MetS), also known as insulin resistance (IR) syndrome, is one of the metabolic disorders with a high risk of negative cardiovascular outcomes, including obesity, hypertension, dyslipidemia, and impaired glucose tolerance (IGT) (7, 8). Recent studies indicated that genes like CTRP7 and SPTAN1 are associated with the occurrence of MetS (9, 10). In addition, various serological indicators were proved to be the potential biomarkers for the diagnosis of MetS (11–13).

The comorbidity burden of SLE with many other diseases has been increasingly reported, such as thyroid diseases, MetS, osteoporosis, cardiovascular diseases, allergic disorders, and some psychiatric problems (14, 15). Male and older patients had higher rates of MetS in SLE, and the most prevalent comorbidities are hypertension (24.6%), dyslipidemia (33.3%), and obesity (35.3%) (15). Disturbances in the homeostasis of metabolism have been demonstrated to exist in some SLE patients, which may be driven by the existence of MetS comorbidity, long-term glucocorticoid usage, and a few other risk factors (14). Notably, despite mounting evidence showed that SLE and MetS are closely connected, these studies tended to take a clinical approach and were unable to reveal the molecular mechanism at the genetic level. Furthermore, research on targeted therapy for comorbidity patients was also minimal.

The gene microarray and scRNA-seq technology provide new insights into the pathogenesis of SLE and MetS, and the bioinformatics analysis helps us to understand the etiology from the genetic perspective. In this study, we performed integrative bioinformatics analysis in combination with machine learning algorithm to identify shared hub genes and pathways in SLE and MetS from GEO database. Additionally, we investigated the correlation between hub genes with immune cells and metabolic pathways in SLE and MetS. In the end, the expression and the location of the most significant hub genes and the related metabolic pathways were verified using scRNA analysis. In general, this might be the first study to establish the shared biomarkers and related metabolic pathways of SLE and MetS, which may offer hints for the exploration of the genetic etiology and combined therapeutic strategy of SLE and MetS comorbidity.

## Materials and methods

### Data selection

**Figure 1:**
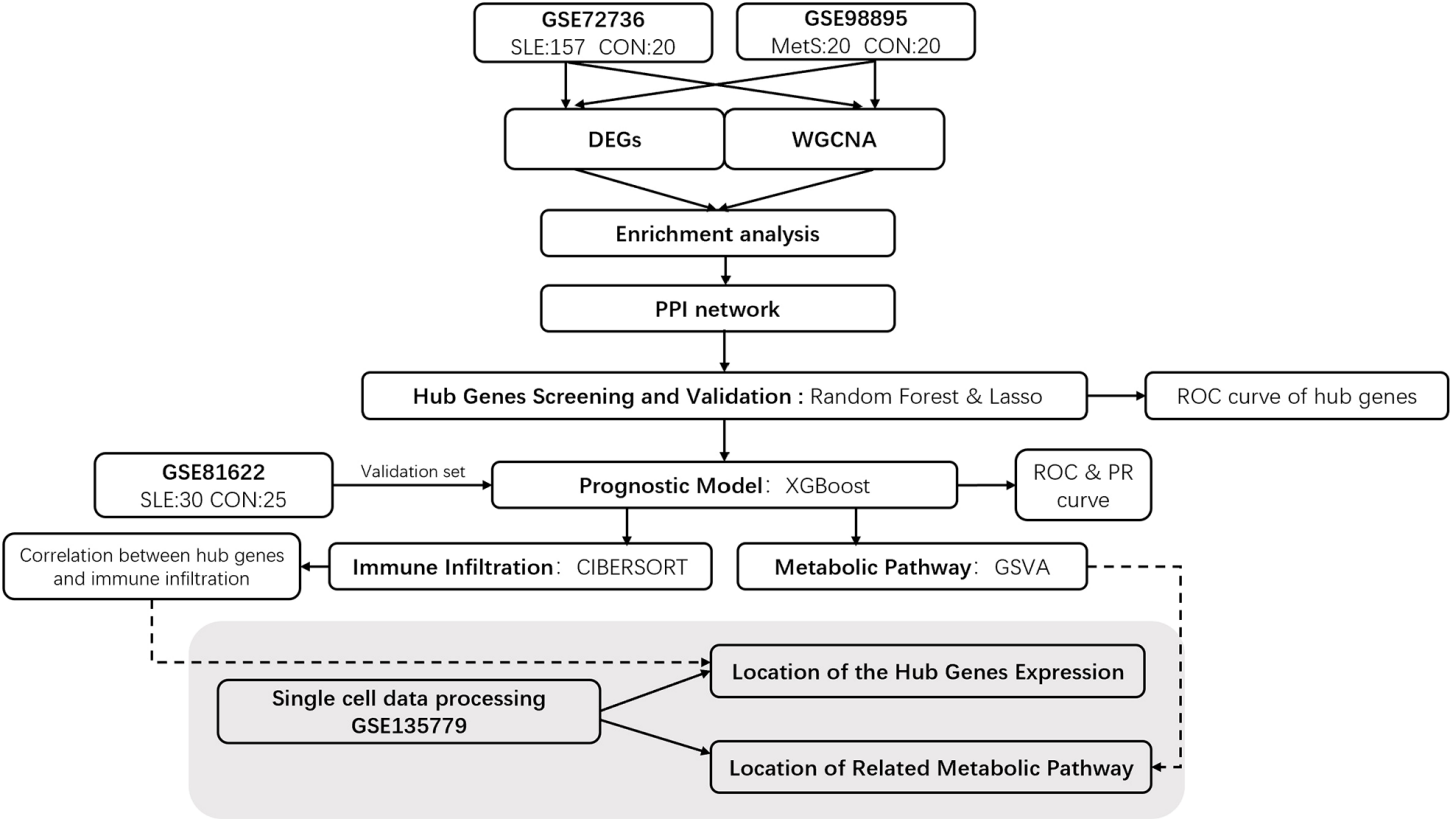
Flowchart of Investigation.

The keywords “lupus” or “SLE” and “Metabolic Syndrome” or “MetS” were used to search gene expression profiles in the GEO database with filter criteria that the samples should be taken from peripheral blood. For SLE, the gene expression profiling by array data sets GSE72326 and GSE81622 were downloaded from the GEO. Data set GSE72326 includes 157 SLE samples and 20 healthy controls samples, and GSE81622 includes 30 SLE samples and 25 healthy controls samples (Platform: GPL10558 Illumina HumanHT-12 V4.0 expression bead chip). For MetS, data set GSE98895 was obtained from the GEO, which contains 20 MetS samples and 20 healthy controls samples. Single-cell sequencing data of SLE patients and healthy controls was procured from the data set of the GSE135779 dataset and was downloaded from GEO, which contains 8 SLE patients and 6 healthy controls. Using Mann-Whitey U test, Chi-square test, and DEGs analysis, we excluded effects of gender and age between patients and healthy controls in those data sets that could introduce a bias in our study. The demographic data of samples in these data sets is provided in **Supplementary Table S1**. The workflow of this investigation is provided in **Figure 1**.

### Identification and visualization of differentially expressed genes

Using the limma R package (16), the GSE72326 and GSE98895 data sets were normalized and analyzed. Adjusted P < 0.05 and |log2FC| ≥ 0.5 were employed as our standard screening criteria for DEGs. Genes were categorized as upregulated or downregulated depending on whether their log2FC value was greater than 0.5 or less than -0.5. To better visualize these DEGs, R software was used to make heatmaps and volcano plots. Heatmaps were created with the pheatmap R package.

### Construction of weighted gene co−expression network analysis

WGCNA was performed on GSE72326 and GSE98895 datasets to screen the gene modules using “pickSoftThreshold” (package WGCNA) (17). The genes ranking in the top 5000 of the median absolute deviation in the corresponding expression matrix were selected for WGCNA. After the missing values and genes with zero variance were filtered out, the extracted values were chosen to build an adjacency matrix based on the scale-free topology criterion (scale-free R2 = 0.9), which was then transformed into a topological overlap matrix (TOM). Next, the average-linkage hierarchical clustering method was used to cluster genes showing similar expression profiles with gene modules. To identify key modules, the minimum module size was determined at 30, and the cut height was set at 0.25. The grey module represented the genes that cannot be merged. Finally, Pearson correlation analysis was performed to calculate the correlation between modules and traits in SLE and MetS.

### Pathway and functional enrichment analysis

We screened feature biomarkers at the intersection of the above DEGs and WCGNA. Based on Metascape (http://metascape.org/gp/index.html#/main/step1), Ontology (GO) term enrichment analysis and Kyoto Encyclopedia of Genes and Genomes (KEGG) pathway analysis were applied for the identification of pathways in which feature genes were significantly enriched of both SLE and MetS (18). The conditions for screening significantly enriched GO terms and KEGG pathways were Min overlap = three and Min Enrichment = 1.5. The enrichment significance threshold was set to an adjusted p-value below 0.05.

### Hub genes screening and validation based on the machine learning algorithm

Random forest algorithm (19) and the least absolute shrinkage and selection operator (LASSO) logistic regression (20) were applied for hub genes screening from the intersection genes of DEGs and WGCNA. To better analysis, we used the sva R package (21) to combine and remove batch effects of GSE72326, GSE81622 (used for validation set) and GSE98895 for further study and performed principal component analysis (**Supplementary Figure S1**). Random forest was performed by the randomForest R package to build a classifier, which compares and ranks the features by importance. LASSO regression was realized by the glmnet R package (22) to reduce data dimensions after the random forest further. In turn, the genes screened by these two algorithms were regarded as hub genes.

### Construction of the prognostic model based on the hub genes

The R package xgboost was used for constructing the Extreme Gradient Boosting (XGBoost) classifier, and the expression values of hub genes were used as eigenvalues for the training of the XGBoost model (23). Firstly, we selected the SLE data set GSE72326 MetS data set GSE98895 as the training sets. Since there is no validation data set available for MetS, we used another SLE data set GSE81622 for validation. The prognostic efficiency was evaluated by receiver operating characteristic (ROC) or precision-recall (PR) curves (24) and their AUC values.

### Correlation analysis of hub gene expression with immune infiltration

CIBERSORT is a deconvolution algorithm widely used to label genomes of different types of immune cells in the microenvironment (25). This study used CIBERSORT to analyze the proportion of 22 immune cells in peripheral blood of GSE72326 and GSE98895. CIBERSORT p-value < 0.05 was included. Pearson correlation coefficient was used to determine the correlation between hub genes and immune-infiltrated cells. Vioplot and pheatmap R packages were used for visualization.

### Correlation analysis of hub gene expression with metabolic pathway

Gene Set Variation Analysis (GSVA) is a non-parametric and unsupervised method for estimating the changes in specific gene sets (26). The activities of the 50 hallmark pathways were quantified with the GSVA R package to find the related metabolic pathways in SLE and MetS. In this part, p < 0.05 was regarded as statistically significant. Pearson correlation coefficient was used to determine the correlation between hub genes and metabolic pathways. Pheatmap R packages were used for visualization.

### Single-cell data processing and clustering

For single-cell sequencing analysis, raw data for GSE135779 were downloaded from GEO, and the package of Seurat (version 4.1.0) was used to process data with R studio (27). ScRNA-seq data quality control was necessarily performed as previously described. Cells were filtered with the criteria of >20% mitochondria-related genes. Based on the variance stabilization transformation (vst), each sample’s first 3000 highly variable genes were analyzed after normalization. The first 3000 highly variable genes screened above were scaled using the function of ScaleData, and the dimension of PCA was reduced using the function of RunPCA. We chose dim = 20 and clustered the cells into 17 cell populations using the FindNeighbors and FindClusters functions. Then the function of RunUMAP was performed for the visualization. For cell population annotation, signatures of CD3E, IL-7R, CCR7, CD4, CD8A, and CCL5 were chosen for T cell annotation; signatures of FOXP3 and IL2RA were chosen for Treg cell annotation; signatures of KLRB1, NKG7, and GNLY were chosen for NK cell annotation; signatures of LYZ, CD14, CD68, S100A9, CD16, FCGR3A, and CD1C were chosen for monocytes annotation; signatures of FCER1A and CST3 were chosen for myeloid DC (m-DC) annotation; signatures of LILRA4 and CLEC4C were chosen for plasmacytoid DC (p-DC) annotation; signatures of MS4A1, CD19, and CD79A were chosen for B cell annotation; and signatures of CD27 was chosen for memory T or B cell demonstration. Furthermore, the VlnPlot function was used to verify the location and expression pattern of potential biomarkers and metabolic pathways in different cell types. The comparisons of the metabolic pathway scores were conducted by Wilcoxon rank-sum test. The visualization of potential biomarkers and metabolic pathways was performed by the FeaturePlot function.

## Results

### Differential expression genes identification in SLE and MetS

**Figure 2:**
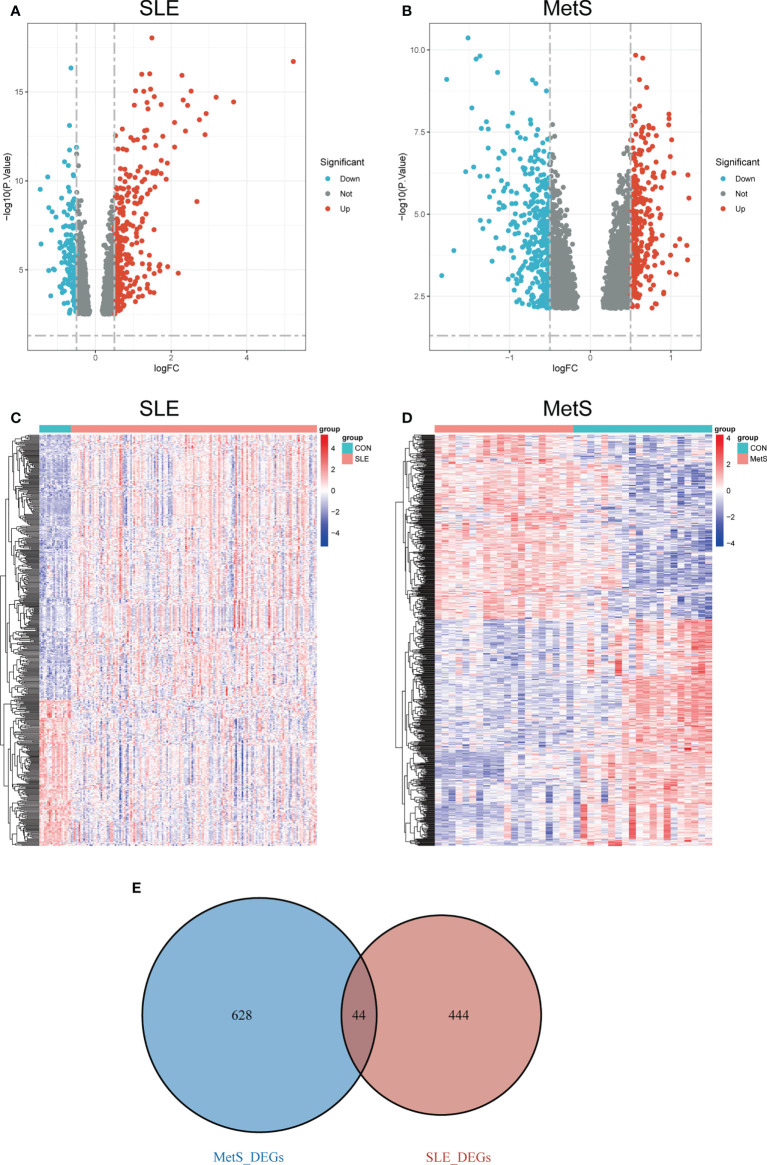
**(A)** Volcano plot of DEGs in GSE72326 |log2FC| > 0.5). **(B)** Volcano plot of DEGs in GSE98895 |log2FC| > 0.5). Red: upregulated; blue: downregulated. **(C)** Heatmap of DEGs in GSE72326. **(D)** Heatmap of DEGs in GSE98895. **(E)** Overlapping DEGs of GSE72326 and GSE98895. Volcano plots showed the genes that are up- or down-regulated in the data sets, with red dots indicating significant up-regulation and blue dots indicating significant down-regulation. Heatmaps exhibited the expression levels of genes in each sample in the data set.

With the limma R package, a total of 488 differential genes (DEGs) were identified based on the SLE dataset GSE72326. The volcano plot shows the identified DEGs, including 314 upregulated and 174 downregulated (**Figure 2A**) using the logFC value. The heatmap demonstrates DEGs (**Figure 2C**). Besides, a total of 672 DEGs were obtained from the MetS dataset GSE98895, among which 301 genes were upregulated and 371were found to be downregulated (**Figure 2B**). Heatmaps of DEGs are shown in **Figure 2D**. A total of 44 overlapping DEGs of GSE72326 and GSE98895 were observed (**Figure 2E**).

### Weighted gene co-expression network analysis of SLE and MetS

**Figure 3:**
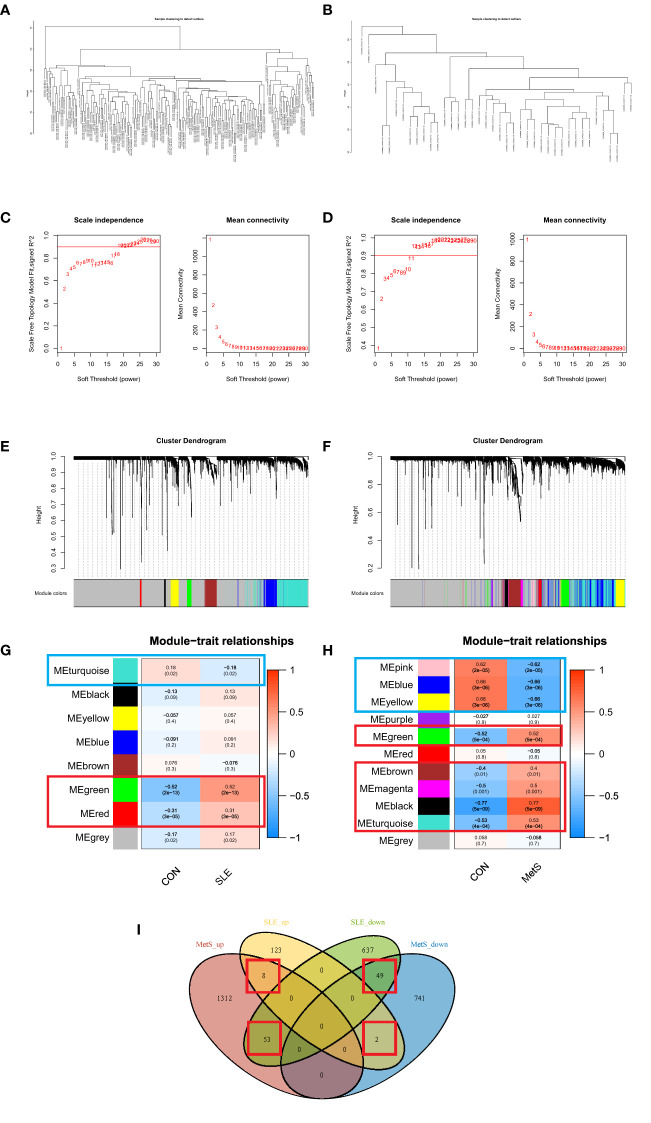
**(A)** Clustering according to the expression level of SLE patients in GSE72326. **(B)** Clustering according to the expression level of MetS patients in GSE98895. Each branch represents a sample in the data sets, and there is no outlier sample in each data set. **(C)** Determination of Soft Threshold power for GSE72326. **(D)** Determination of Soft Threshold power for GSE98895. When scale-free distribution is reached, the optimal soft-power value for GSE72326 was 19 while for GSE98895 it was 11. **(E)** Origin and merged modules displaying under the clustering tree for GSE72326. **(F)** Origin and merged modules displaying under the clustering tree for GSE98895. Cluster dendrograms showed the clustering process of the gene modules **(G)** Heatmap of the correlation between module eigengenes and the occurrence of SLE. **(H)** Heatmap of the correlation between module eigengenes and the occurrence of MetS. **(I)** Overlapping module genes of GSE72326 and GSE98895.

We performed WGCNA to explore the correlation between clinical traits and genes. All samples were clustered in the GSE72326 and GSE98895 datasets, and none of the samples was eliminated (**Figures 3A, B**). According to the WGCNA methodology, the optimal soft-power value for GSE72326 was 19 while for GSE98895 it was 11 (**Figures 3C, D**). A total of 8 modules were identified in GSE72326, and 11 were identified in GSE98895. Afterwards, the correlations between the module and clinical traits were calculated. The green and red modules had the strongest positive relation with SLE (r = 0.52 and 0.31), while the turquoise module had the strongest negative relation (r = 0.18) in the GSE72326 database (**Figures 3E, G**). For MetS, the green, magenta, black, and turquoise modules showed the strongest positive correlation (r = 0.52, 0.4, 0.5, 0.77, and 0.53), whereas pink, blue, and yellow modules had the strongest negative correlation (r = 0.62, 0.66, and 0.66) in the GSE98895 database (**Figures 3F, H**). A total of 112 overlapped module genes of GSE72326 and GSE98895 were observed (**Figure 3I**).

### Enrichment analysis and PPI network construction

There were 112 genes shared by the SLE and MetS modules. Moreover, for DEGs, 44 shared genes were found. There were only 3 overlapping genes of DEGs and WGCNA module genes. Firstly, the modules screened from WGCNA contain a cluster of genes with similar expression profiles, which may not cover the full range of DEGs or even differ a lot from DEGs. Secondly, some DEGs did not consist of modules with other similar genes, which may also be critical for the development of disease. In order not to cause the omission, we combined DEGs and modules genes together to be the candidate genes for the following analyses.

**Figure 4:**
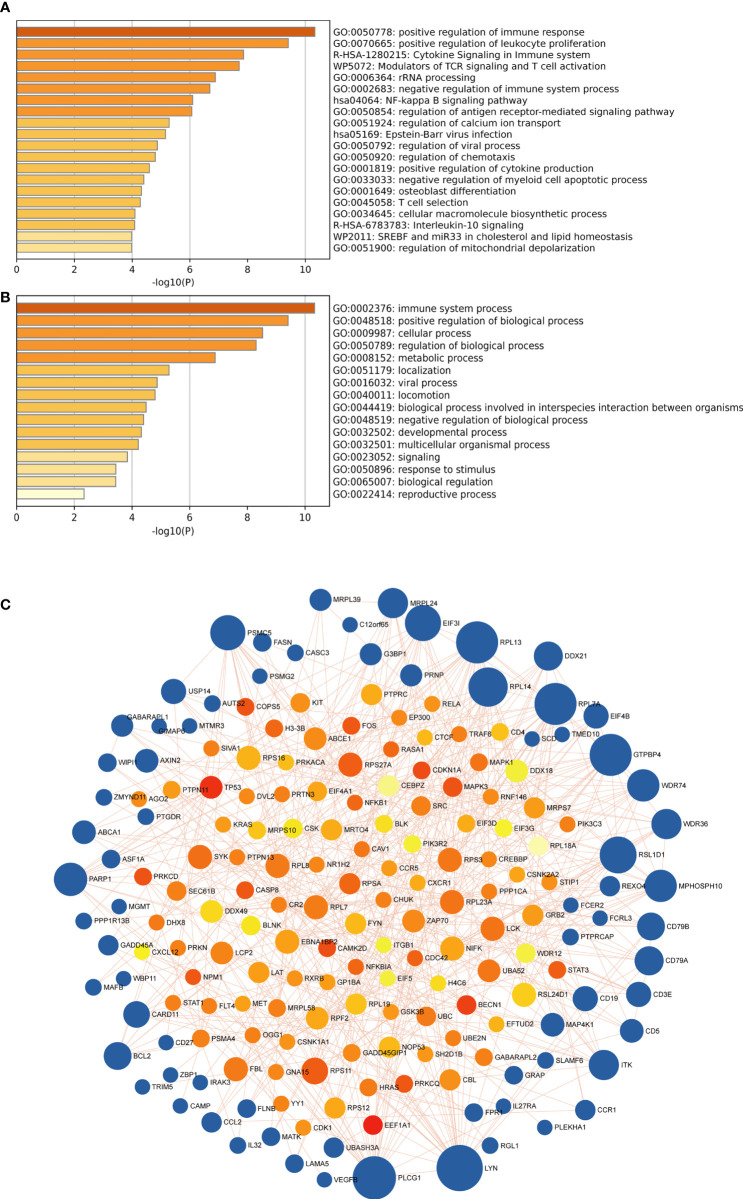
**(A)** the top 20 enriched clusters across feature genes, colored by p-values. **(B)** The top-level Gene Ontology biological processes colored by p-values. The darker the color, the stronger the enrichment of the gene in that pathway. **(C)** The PPI network of feature genes. Each blue node represents a gene, and each other node represents a protein. Different sizes indicate the core degree of genes in the PPI network, whereas a bigger size indicates more importance in the network.

Then we got 153 candidate genes, which may be the important players involved in the pathogenesis of both SLE and MetS and potentially possess a shared molecular mechanism. Therefore, to further explore the signaling pathway associated with those genes involved in SLE and MetS, pathways and functional enrichment analyses were carried out on the basis of GO, including biological processes, cellular components, and molecular functions, and the KEGG comprised significantly enriched signaling pathways. According to the results of GO/KEGG enrichment analyses, those genes were significantly involved in the positive regulation of immune response, positive regulation of leukocyte proliferation, cytokine signaling in the immune system, modulators of TCR signaling and T cell activation, rRNA processing, and NF-kappa B signaling pathway, et al. (**Figure 4A**). Meanwhile, we found those feature genes were not only enriched in immunological but also in metabolic-related terms (**Figure 4B**). Moreover, we constructed a protein-protein interaction (PPI) network based on the STRING database. A total of 188 nodes and 445 edges were obtained with a combined score >0.8, as shown in **Figure 4C**. The top3 significant genes with the highest ranking were LYN proto-oncogene (*LYN*), phospholipase C, gamma 1 (*PLCG1*), and ribosomal protein L13 (*RPL13*).

### Identification and validation of potential shared hub genes by random forest and LASSO

**Figure 5:**
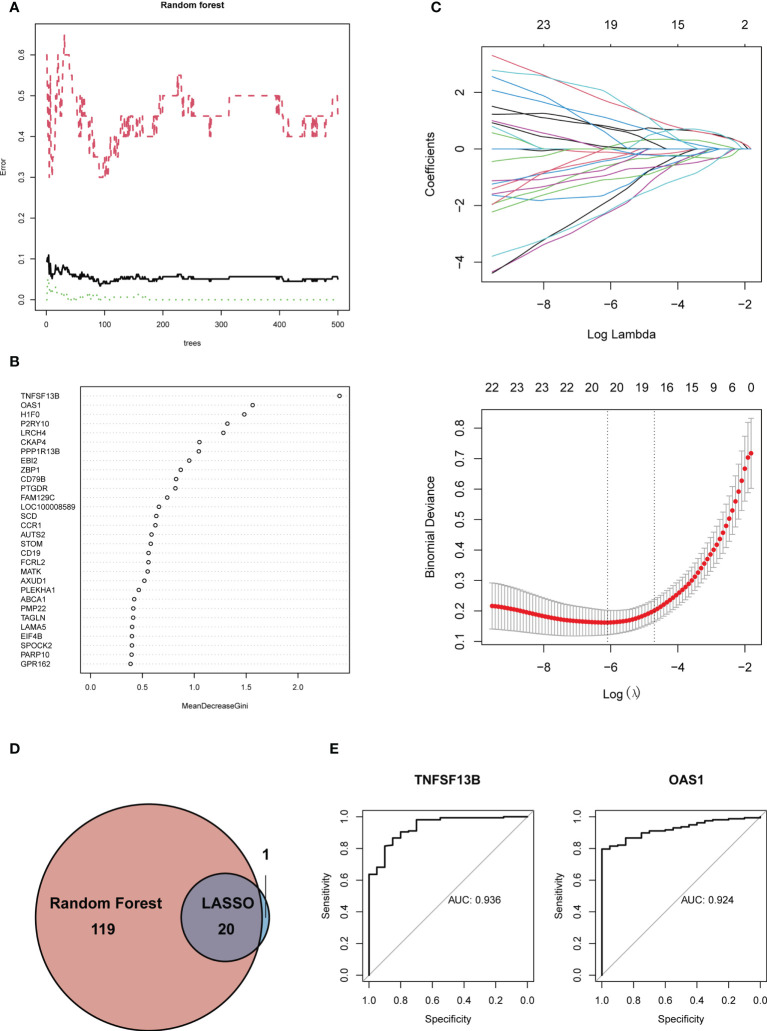
**(A)** Gene selection *via* Random Forest algorithm. **(B)** The top 30 significant genes recognized from Random Forest. MeanDecreaseGini showed the rank of genes in accordance with their relative importance. **(C)** The performance in of ten-time cross-verification for tuning parameter in selection least absolute shrinkage and selection operator (LASSO). Each coefficient curve in the upper picture represents a single gene. The solid vertical lines in another picture represent the partial likelihood deviance SE, and the number of genes (n = 20) corresponding to the lowest point of the curve is the most suitable for LASSO. **(D)** The intersected genes of these two algorithms were selected. **(E)** ROC curves of TNFSF13B (AUC = 0.936, 95%CI 0.885-0.986) and OAS1 (AUC = 0.924, 95%CI 0.883-0.965).

To further screen the hub genes with the most diagnostic values, we selected the foremost characteristics based on machine-learning algorithms. Random Forest analysis and LASSO regression analysis were carried out in succession. Random forest was used to identify 139 genes from 153 feature genes (**Figure 5A**). **Figure 5B** showed the top 30 significant genes, with TNF Superfamily Member 13b (*TNFSF13B*) and 2’-5’-Oligoadenylate Synthetase 1, and H1 histone family, member 0 (*H1F0*) having the highest MeanDecreaseGini. At the same time, 21 genes were screened from DEGs by LASSO logistic regression (**Figure 5C**). By overlapping genes screened from Random Forest and LASSO, we eventually obtained 20 shared hub genes, which were considered to carry the maximum diagnostic value (**Figure 5D**). Moreover, we validated the diagnostic prognostic efficacy of each shared hub genes through ROC curve (**Table 1**, **Supplementary Figure S2**), with *TNFSF13B* (AUC = 0.936) and *OAS1* (AUC = 0.924) having the highest AUC (**Figure 5E**).

**Table 1:** AUC of 20 hub genes.

Gene	AUC of ROC	95% CI
SCD	0.744	0.643–0.846
OAS1	0.924	0.883–0.965
LOC100008589	0.816	0.721–0.911
TNFSF13B	0.936	0.885–0.986
WIPI1	0.703	0.577–0.829
C21orf51	0.571	0.434–0.708
H1F0	0.885	0.818–0.952
STOM	0.781	0.679–0.883
FCRLA	0.839	0.767–0.912
TAGLN	0.748	0.634–0.862
FAM129C	0.859	0.792–0.925
CD79B	0.86	0.799–0.921
AUTS2	0.859	0.787–0.931
NOSIP	0.719	0.616–0.822
LOC728643	0.72	0.583–0.857
PMP22	0.805	0.719–0.890
EBI2	0.827	0.715–0.939
PPP1R13B	0.867	0.792–0.941
LRCH4	0.721	0.592–0.850
LAMA5	0.851	0.785–0.917

### Construction of prognostic model based on XGBoost

**Figure 6:**
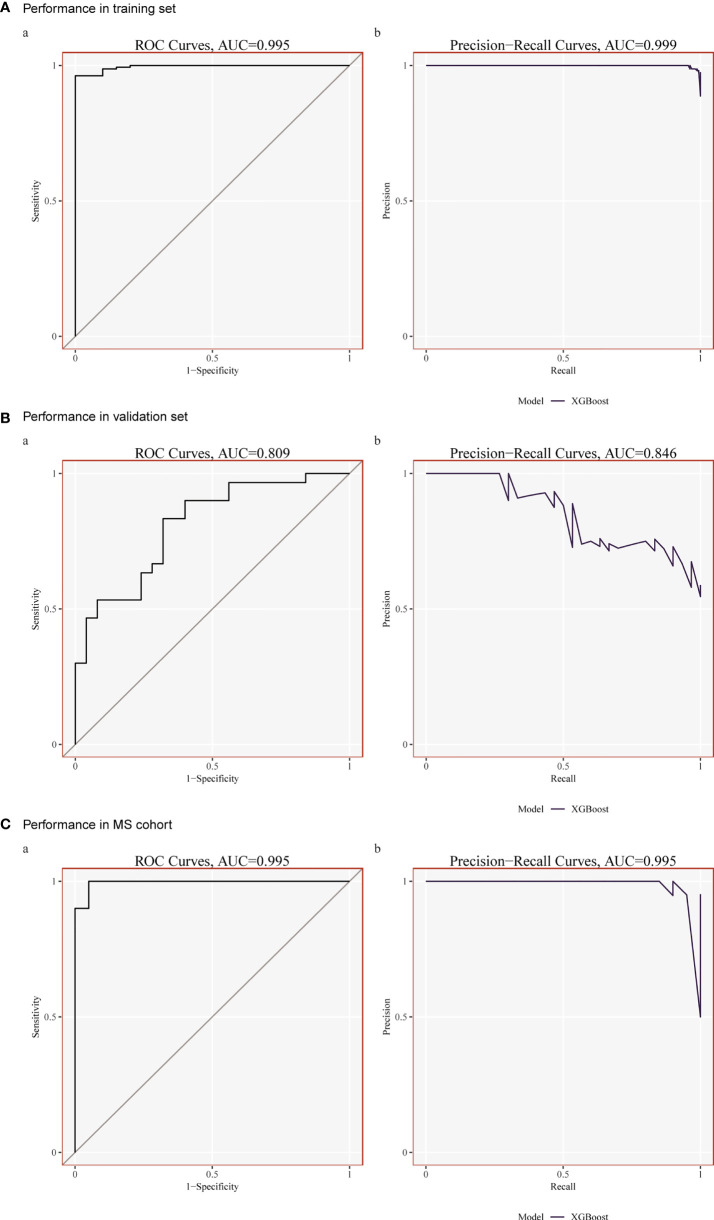
**(A)** Performance in the training set (GSE72326) using XGBoost. **(B)** Performance in the Validation set (GSE81622) using XGBoost. **(C)** Performance in the training set (GSE98895) using XGBoost.

Although each shared hub gene can be employed as an auxiliary diagnostic or predictive biomarker, we prefer to develop a comprehensive prognostic model to increase the effectiveness of diagnosing or predicting diseases. Therefore, we utilized machine learning to ascertain whether these 20 hub genes can construct a comprehensive prognostic model. XGBoost algorithm, a popular algorithm in machine learning classifiers that has demonstrated excellent performance (28), was selected for generating the model based on those 20 hub genes. In order to improve the model, XGBoost calculates negative gradients and uses them to find problems. Training and testing sets were used to confirm the effectiveness and dependability of the prognostic model. We chose one SLE dataset (GSE72326) for training and another SLE dataset (GSE81622) for validation. The performance of the training set showed that the AUC of ROC and Precision-Recall curve were 0.995 and 0.999 (**Figure 6A**), and the AUC of the validation set were 0.809 and 0.846 (**Figure 6B**), respectively, indicating that this prognostic model had good performance in distinguishing SLE patients from healthy controls. Meanwhile, to verify whether it is able to identify MetS patients, we used the MetS dataset (GSE98895) to train the model based on the same shared hub genes, and the result showed its equally well performance (**Figure 6C**).

### Immune cell infiltration and correlation with shared hub genes

The enrichment analysis revealed that immunity plays an essential role in developing this comorbidity, so we investigated whether distinct patterns of immune infiltration could be discerned based on the 22 types of immune cells by the CIBERSORT method. First, we evaluated the composition of the immune cell infiltrate in peripheral blood of the SLE data set (GSE72326) and MetS data set (GSE98895).

**Figure 7:**
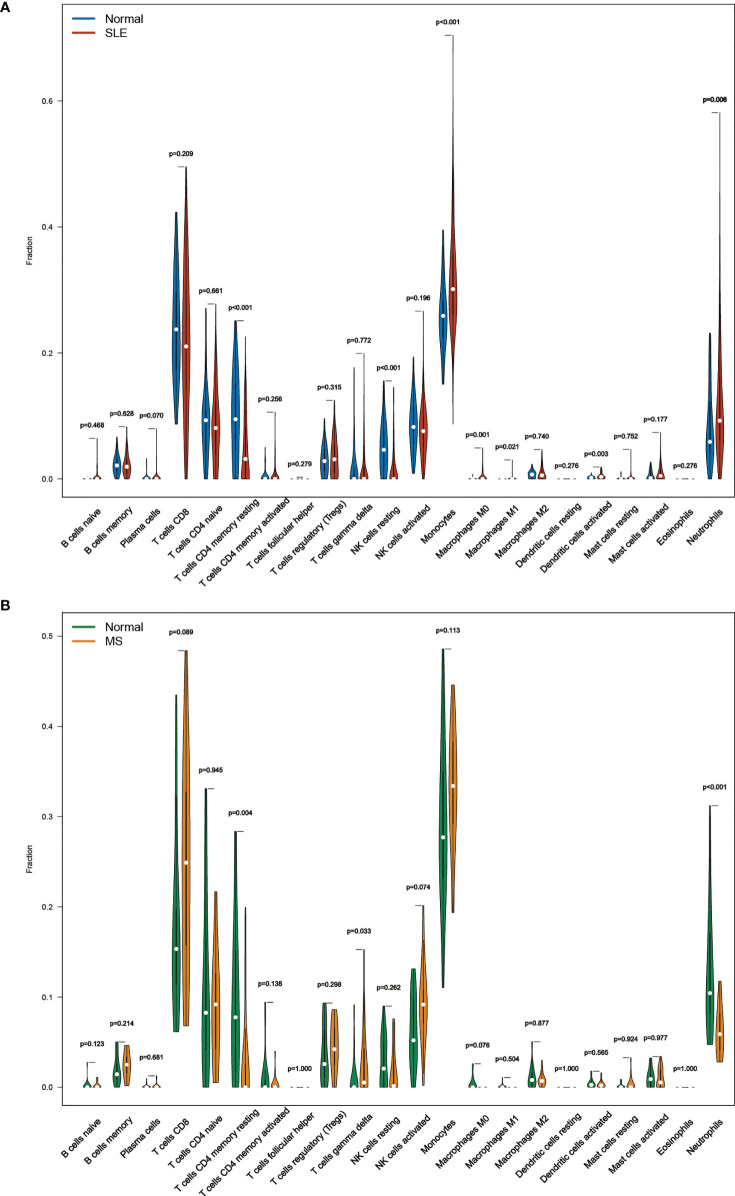
**(A)** The composition of the immune cell infiltrate in SLE. **(B)** The composition of the immune cell infiltrates in MetS.

The Violin diagram demonstrated significant differences between SLE and control samples in monocytes, NK cells, macrophages, dendritic cells, neutrophils, and CD4^+^ memory T cells populations (**Figure 7A**). Compared with the normal sample, resting CD4^+^ memory T cells and resting NK cells were considerably decreased in the SLE sample, while monocytes, M0 macrophages, M1 macrophages, activated dendritic cells, and neutrophils were significantly increased. We also performed CIBERSORT in the MetS data set (GSE98895). The result showed that resting CD4^+^ memory T cells and neutrophils were decreased in patients while gamma delta T cells increased (**Figure 7B**).

**Figure 8:**
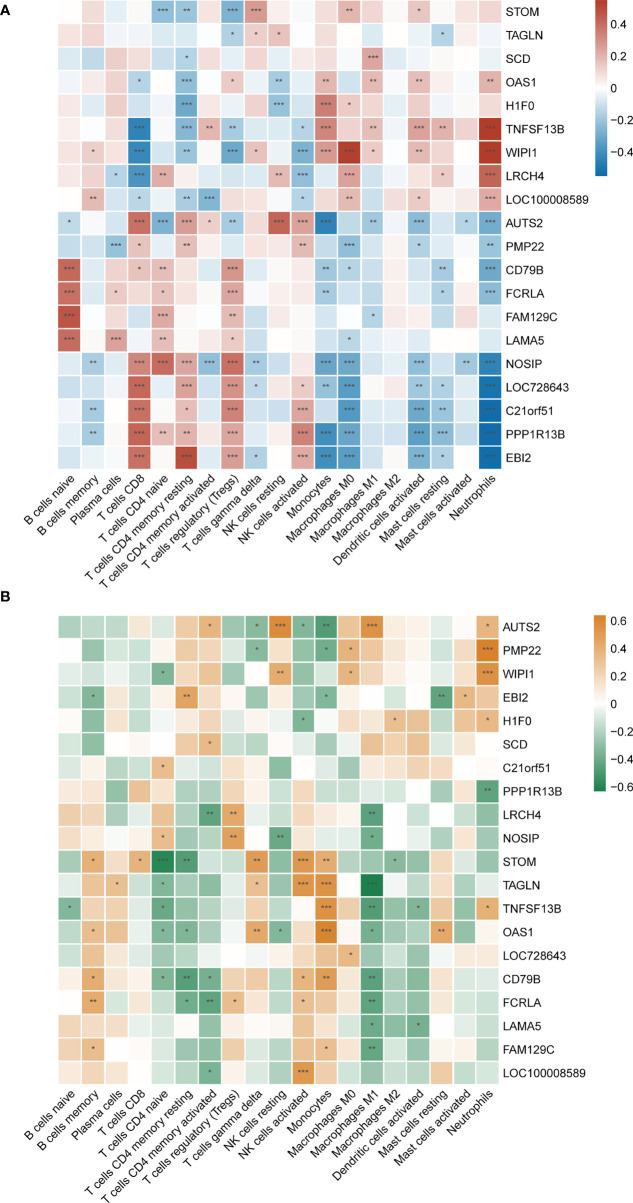
**(A)** Correlation matrix between immune cell proportions and shared hub genes in SLE. Red represents for positive correlation, while blue for negative correlation. **(B)** Correlation matrix between immune cell proportions and shared hub genes in MetS. Orange represents for positive correlation, while green for negative correlation. Asterisks represent levels of significance (*p < 0.05, **p < 0.01, ***p < 0.001).

However, common differences in immune cell composition ratios are only one aspect of the common pathogenesis of SLE and MetS. We still need to confirm whether these 20 shared hub genes are associated with immune infiltration in the peripheral blood, and if so, specifically which immune cells they are associated with, as well as to identify their commonalities. Therefore, Pearson correlation analysis was used to investigate the correlations between shared hub genes with immune cells in SLE. We observed that monocytes, M0 macrophages, activated dendritic cells, and neutrophils had a similar significant positive correlation with *TNFSF13B*, *WIPI1*, and *OAS1*, and a significantly negative relationship with *AUTS2*, *PPP1R13B*, *EBI2*, and *NOSIP*. Additionally, resting NK cells were positively correlated with *AUTS2* and *LRCH4* but negatively correlated with *OAS1* and *H1F0* (**Figure 8A**).

As mentioned earlier, since both *TNFSF13B* and *OAS1* possessed the highest diagnostic potency, we observed that neutrophils, monocytes, activated dendritic cells, activated CD4^+^ memory T cells, M1 macrophages, and resting mast cells had a significantly positive correlation with *TNFSF13B*, while negatively correlated with CD8+ T cells, resting CD4+ memory T cells, regulatory T cells (Tregs), and NK cells. For *OAS1*, a similar correlation was observed with those cells in front, with the exception of activated memory T cells, Tregs, and resting mast cells. Also, we observed some relationships between hub genes and immune cells in MetS (GSE98895) (**Figure 8B**), suggesting that immune-related cells were also involved in the pathogenesis of MetS. Both *TNFSF13B* and *OAS1* had a significantly positive correlation with monocytes, while negatively related to naïve CD4^+^ T cells and M1 macrophages. Generally speaking, there is a consistent correlation between these two genes and monocytes in SLE and MetS.

### Metabolic pathway involvement and correlation with shared hub gene

**Figure 9:**
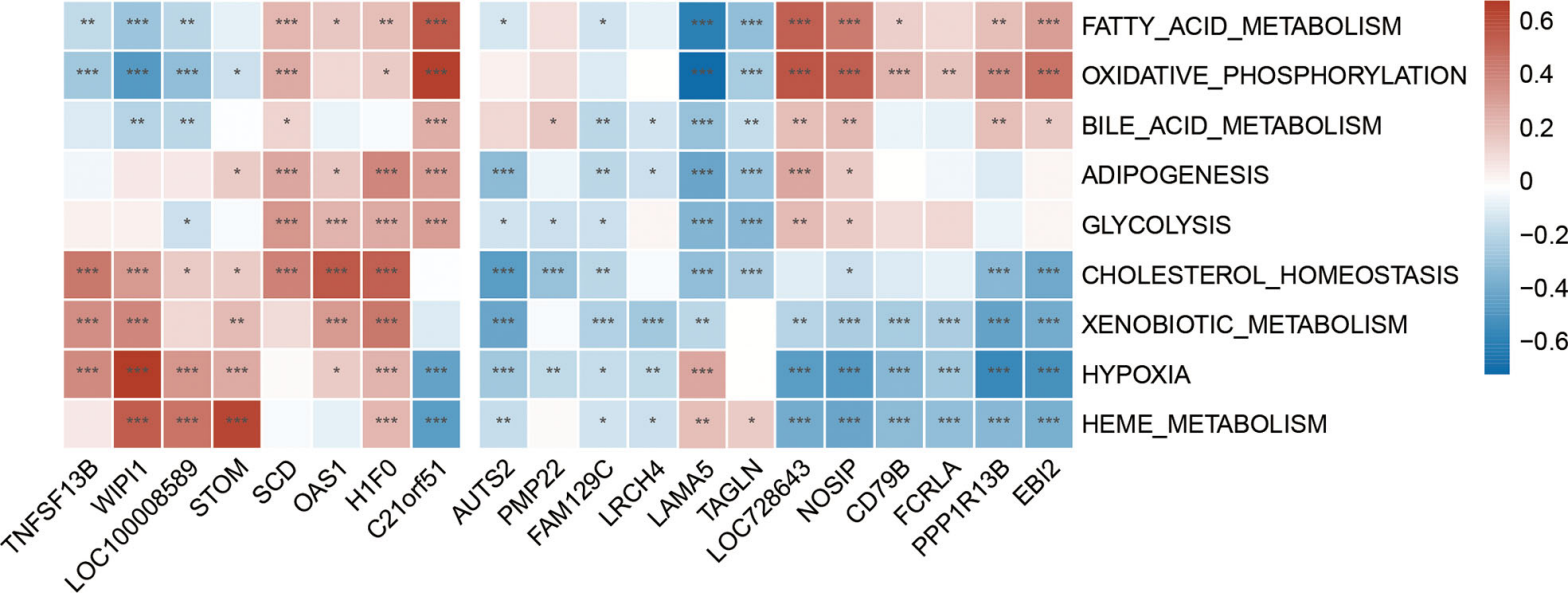
Correlation matrix between metabolic pathways and shared hub genes in SLE. The left part showed those up-regulated hub genes, and the right part showed the down-regulated hub genes. Red represents for positive correlation, while blue for negative correlation. Asterisks represent levels of significance (*p < 0.05, **p < 0.01, ***p < 0.001).

The GSVA result of the relevant metabolic pathway is presented in the heatmap (**Figure 9**). 9 in 50 hallmark pathways were involved in the metabolic process, and we completed Pearson correlation analysis to determine the relationship between our hub genes and those nine metabolic pathways. Overall, this result suggested that cholesterol homeostasis, xenobiotic metabolism, hypoxia, and heme metabolism were highly and consistently correlated with hub genes. In addition, those four pathways were positively correlated with up-regulated hub genes and negatively with down-regulated hub genes. In other words, those four pathways might be activated in SLE and MetS. Focusing on *TNFSF13B* and *OAS1* as they held the highest diagnostic performance, we found these two hub genes were highly positively correlated with cholesterol homeostasis and xenobiotic metabolism in SLE.

### Single-cell analysis for the location of hub genes

**Figure 10:**
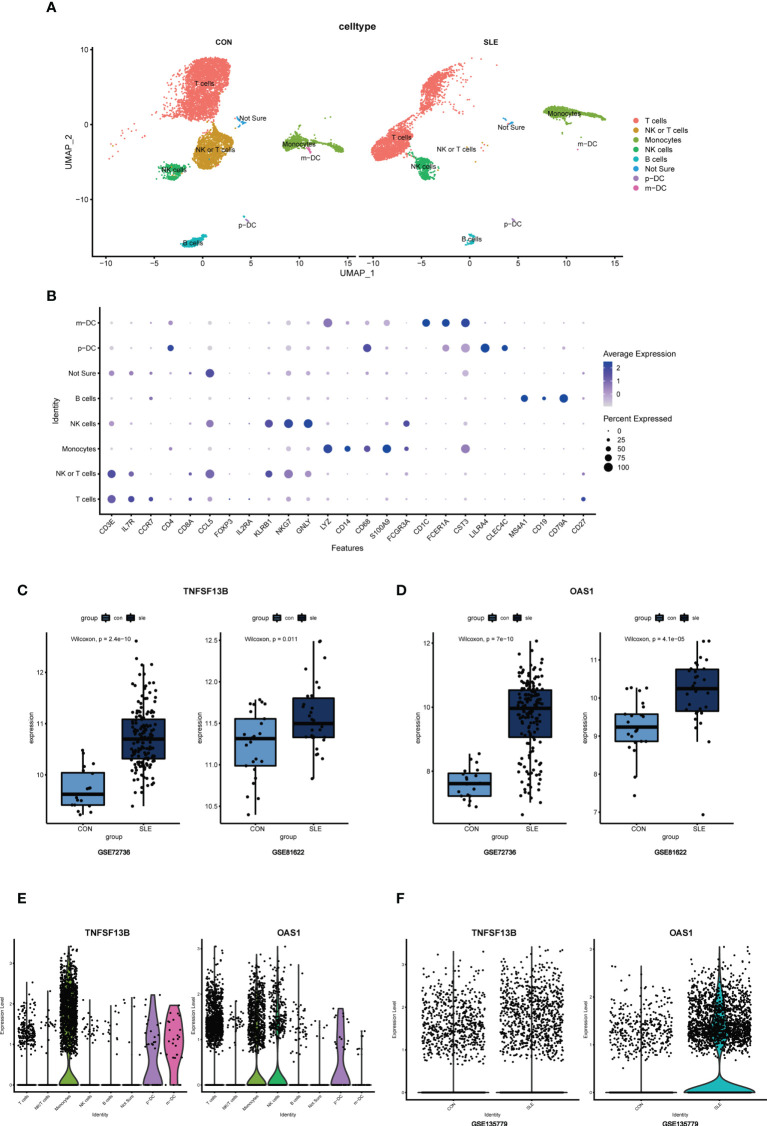
**(A)** UMAP visualization of the 11178 cells in the single-cell RNA seq dataset GSE135779; Different colors indicate distinct clusters; m-DC: myeloid dendritic cells; p-DC: plasmacytoid dendritic cells. **(B)** Dot plot of cell population annotation based on different signatures. **(C)** Boxplots of TNFSF13B expression levels in GSE72326 and GSE81622; Comparison was conducted by Wilcoxon rank-sum test. **(D)** Boxplots of OAS1 expression levels in GSE72326 and GSE81622; Comparison was conducted by Wilcoxon rank-sum test. **(E)** The expression level of TNFSF13B and OAS1 in 8 clusters of cells in GSE135779. **(F)** The expression level of TNFSF13B and OAS1 in controls and SLE patients in GSE135779.

In addition to transcriptomics analysis, we evaluated the immune microenvironment of peripheral blood using scRNA-seq data GSE135779. After QC (**Supplementary Figure S3**), we clustered 11178 cell populations into 17 clusters (**Supplementary Figure S4**). Using genes CD3E, IL-7R, CCR7, CD4, CD8A, CCL5, FOXP3, IL2RA, KLRB1, NKG7, GNLY, LYZ, CD14, CD68, S100A9, CD16, FCGR3A, CD1C, FCER1A, CST3, LILRA4, CLEC4C, MS4A1, CD19, CD79A, and CD27, we classified 17 cell clusters into 7 cell populations, including 6376 T cells, 1676 monocytes, 820 NK cells, 449 B cells, 40 p-DCs, 26 m-DCs, and 60 cells that are not ultimately defined (**Figures 10A, B**). Results showed that the cell clusters comprising T cells, monocytes, and NK cells were not identical in SLE and control samples, suggesting that the subpopulations of these cells may be different, following the CIBERSORT results above.

Since *TNFSF13B* and *OAS1* are the two with the highest diagnostic efficacy among hub genes, we selected them for further study to assess their expression and localization in PBMC between SLE and normal samples. The expression level of *TNFSF13B* and *OAS1* were both elevated in GSE72326 and GSE81622 (**Figures 10C, D**), which was verified in scRNA-seq data GSE135779 (**Figure 10F**). It has been well recognized that *TNFSF13B* was mainly expressed by monocyte clusters in SLE, which was consistent with our result of scRNA analysis (**Figure 10E**). For gene *OAS1*, however, we had very limited knowledge of the cells in which it was mostly expressed in SLE. Thus, we further examined *OAS1* in single-cell populations.

**Figure 11:**
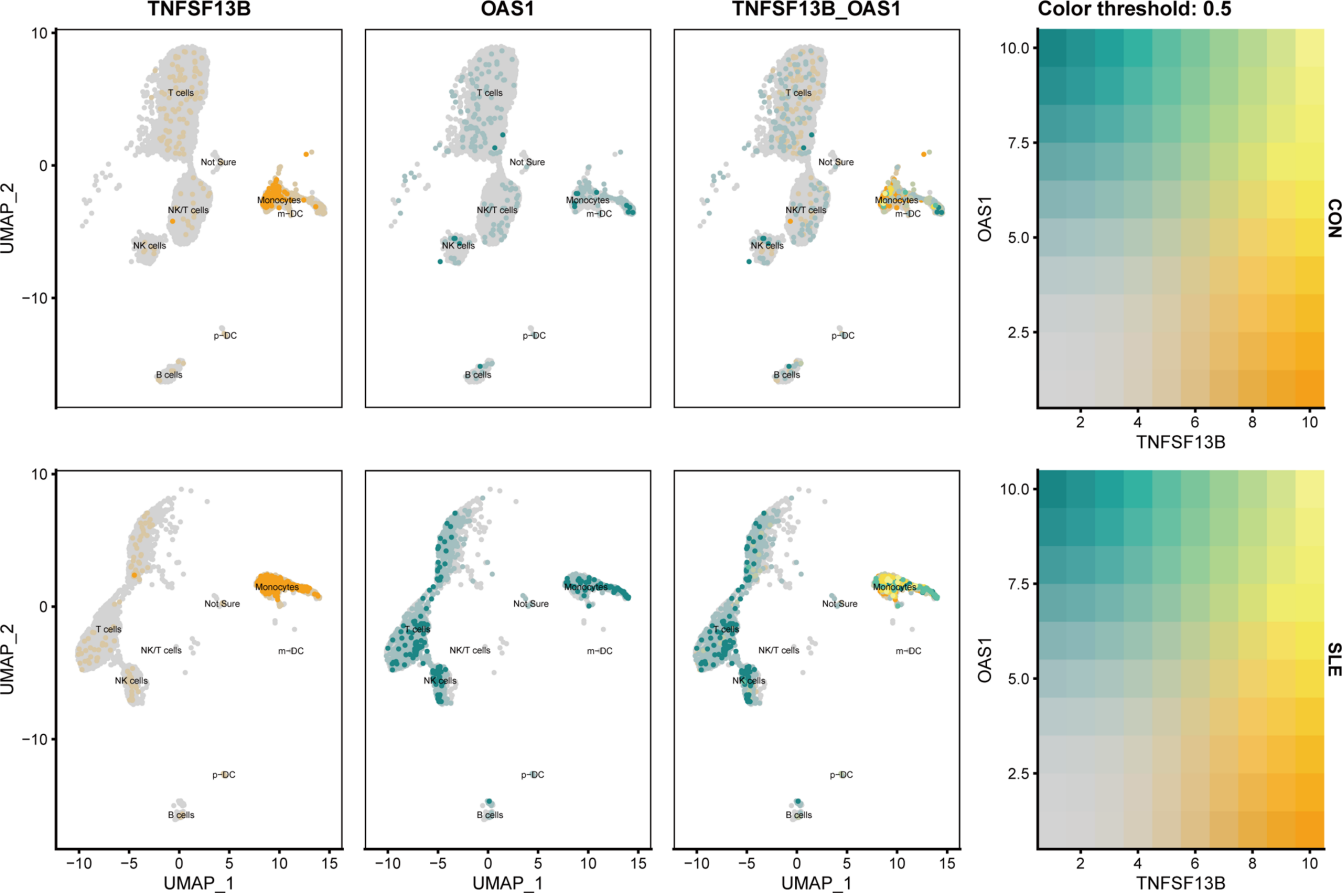
UMAP visualization of TNFSF13B and OAS1 expression in controls and SLE patients in GSE135779. Yellow dots indicated TNFSF13B expression, and cyan dots indicated OAS1.

As noticed, monocytes, NK cells, and T cells showed high expression of *OAS1* (**Figure 10E**). Using UMAP for the visualization, we found that the expression level of *OAS1* was upregulated and primarily enriched in monocytes, which was similar to *TNFSF13B* (**Figure 11**). In other words, the core cell type that was strongly associated with the expression of both *TNFSF13B* and *OAS1* was represented by monocytes.

### Single-cell analysis for the location of metabolic pathways

**Figure 12:**
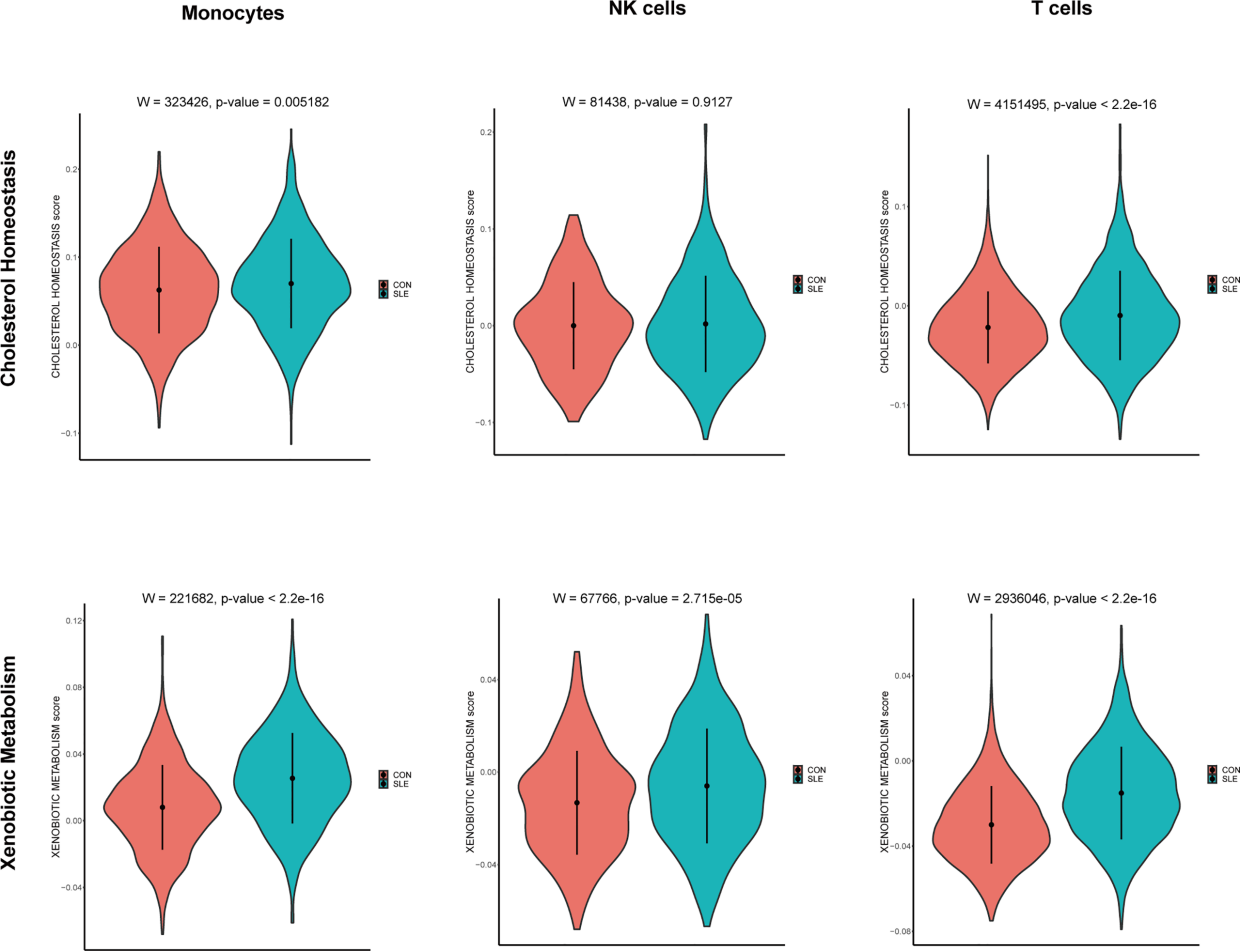
Violin plots of the cholesterol homeostasis and xenobiotic metabolism levels in monocytes, NK cells, and T cells of controls and SLE; Comparison was conducted by Wilcoxon rank-sum test.

**Figure 13:**
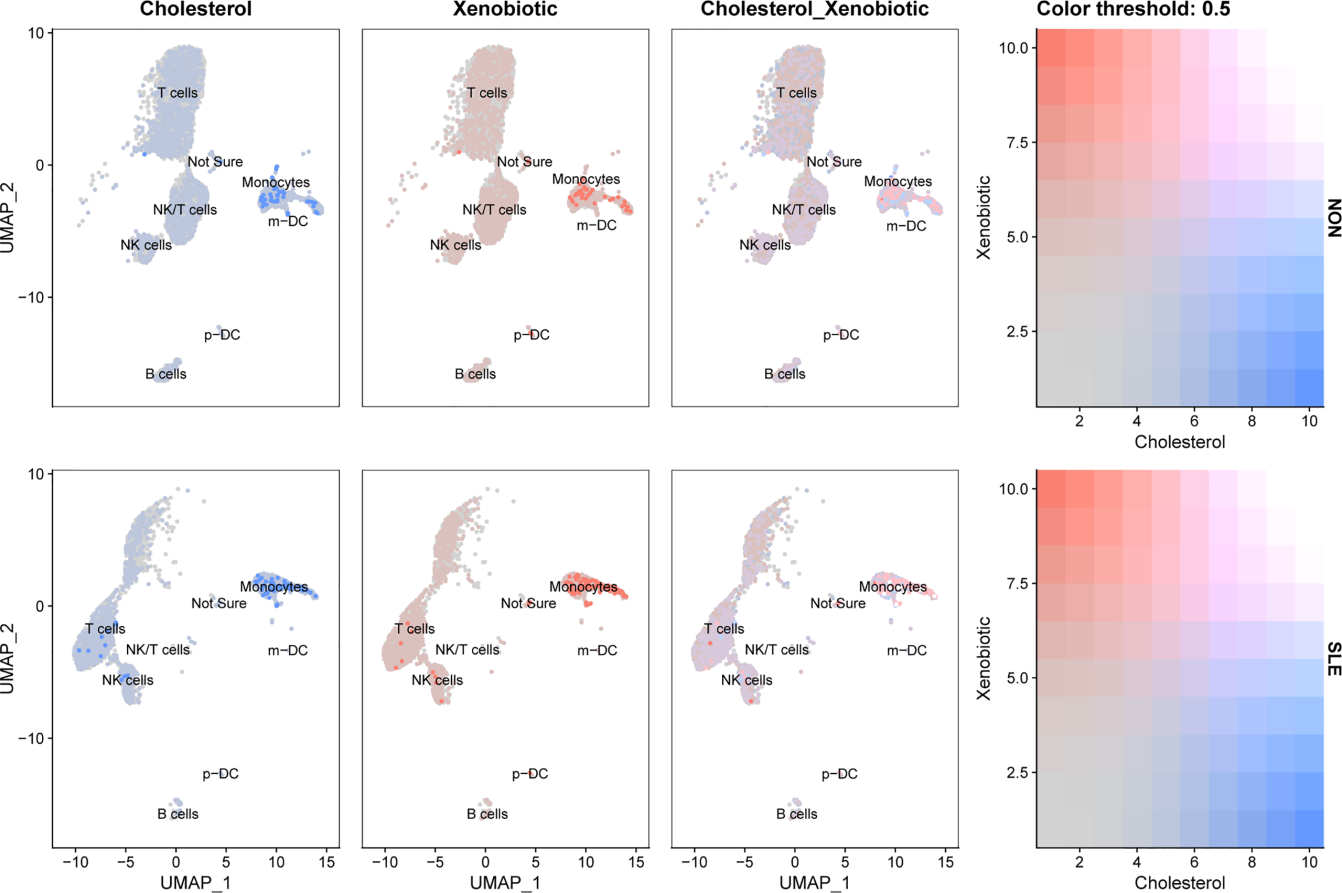
UMAP visualization of cholesterol homeostasis and xenobiotic metabolism scores in controls and SLE patients in GSE135779. Blue dots indicated cholesterol homeostasis, and pink dots indicated xenobiotic metabolism.

According to the front results, *TNFSF13B* and *OAS1* were highly positively correlated with cholesterol homeostasis and xenobiotic metabolism pathways and mainly expressed by monocytes, NK cells, and T cells. Therefore, to investigate whether the expression level of these two metabolic pathways in those three types of immune cells was different in SLE, the comparison of cholesterol homeostasis and xenobiotic metabolism pathways in SLE and healthy controls was performed. We discovered those two metabolic pathways were increased in those three kinds of immune cells with significant differences (p < 0.05), except cholesterol homeostasis in NK cells (**Figure 12**). Next, to further determine which types of immune cells was dominant, we use UMAP for visualization, and those two metabolic pathways were observed to be significantly concentrated in monocytes, which appeared to be accompanied by the expression of *TNFSF13B* and *OAS1* (**Figure 13**) indicating that monocytes may play a vital role in these two kinds of metabolic disorders in the peripheral blood of SLE patients.

## Discussion

Metabolic disorders are often present in SLE patients, and the high incidence of SLE and MetS comorbidity has been increasingly documented (15). As an effective predictor of cardiovascular morbidity and mortality, MetS has been confirmed to predispose SLE patients to a range of cardiovascular events, as well as chronic kidney disease and diabetes (29). Moreover, since the proinflammatory cytokines are a common underlying mechanism of both SLE and obesity, MetS may also act as a trigger and contribute to the accumulation of chronic systemic inflammation and disease activity of SLE (29). Previous intravenous methylprednisolone use, male, older age, higher ESR, higher C3 levels and higher serum creatinine levels are regarded as risk factors for MetS in SLE patients (30). These characteristics, however, were only examined at the clinical or serological levels; the genetic level is still a mystery. Additionally, since altering one’s lifestyle and taking medications like metformin or hydroxychloroquine only had little effectiveness in reducing insulin resistance in cases of SLE and MetS cooccurrence (29), therapies that target specific genes and pathways are urgently needed to be discovered. In addition, clinical data indicated that the main obstacle to target treatment of SLE patients is its heterogeneity in the involvement of different cell types. The clinical heterogeneity of SLE and its related involvement of different cell types has made it challenging to design accurate therapy (31). Therefore, finding the major cell types involved in the pathogenesis of SLE and MetS comorbidity and exploring a cell-specific molecular program is of vital importance.

In this study, to explore whether SLE and MetS have some genetic and molecular mechanism similarities, we performed integrative bioinformatics analysis in combination with machine learning algorithms to identify shared hub genes and pathways in SLE and MetS. Firstly, we analyzed shared DEGs and co-expression modules of SLE and MetS, which lack relevant reports. In order to try not to miss genes associated with the development of SLE and MetS comorbidity, we combined DEGs and WGCNA module genes together, and 153 shared candidate genes were found. Enrichment analyses suggested that those candidate genes were associated with both immune-relate and metabolic-related signaling pathways. Next, to further screened shared hub genes, machine learning approaches were applied. The intersection of Random Forest and LASSO was considered the shared hub genes of SLE and MetS, and the diagnostic efficacy of each shared hub gene was further certificated. TNF Superfamily Member 13b (TNFSF13B) and 2’-5’-Oligoadenylate Synthetase 1 (OAS1) exerted the best diagnostic performance with the highest AUC.

Even though each shared hub gene can be used as a predictive biomarker, the differential expression levels of individual genes may not only present in SLE or MetS, making diagnosis or prediction based on individual genes less precise. Thus, we prefer to construct a comprehensive prognostic model to improve the accuracy of disease diagnosis or prediction using the XGBoost algorithm. The AUC of the training and validation sets indicated that these 20 genes were reliable for prognosing SLE and MetS comorbidity.

In addition, to further investigated the correlation between hub genes with immune cells and metabolic pathways in SLE and MetS, we employed CIBERSORT immune infiltrates analysis and GSVA metabolic pathway analysis, and found some correlations between hub genes with 22 immune cells and nine metabolic pathways. *TNFSF13B* and *OAS1* exerted a significantly positive relationship with monocytes in both SLE and MetS. Additionally, Cholesterol homeostasis and xenobiotic metabolism pathways correlated remarkably with *TNFSF13B* and *OAS1*.

In the end, scRNA-seq analyses were performed to verify the expression and the location of the most significant hub genes and the related metabolic pathways in specific cell types. However, due to the lack of MetS-associated single-cell data from PBMC, we applied only the SLE single-cell dataset for analysis. Finally, we found that these two central genes and metabolic pathways were upregulated and significantly enriched in monocytes.

As one member of the tumor necrosis factor (TNF) ligand family, the cytokine coded by *TNFSF13B* is also known as B cell activating factor (BAFF) and B lymphocyte stimulator (BLyS) (32, 33). BAFF has been shown to be commonly overexpressed in SLE, which plays a vital role in the proliferation and differentiation of B cells and is strongly involved in the pathogenesis of SLE (34). A recent study revealed that *TNFSF13B* expression was elevated in SLE and highly positively correlated with monocytes (31), which was consistent with our results of CIBERSORT and scRNA analyses. Belimumab, the only effective biological therapy that targets BAFF, has been authorized for clinical use in SLE (35). Notably, since belimumab has been widely used in the clinical treatment of SLE and has exerted efficient and safe therapeutic effects, our results suggested that this drug may also be appropriate for the combination treatment of SLE and MetS comorbidity.

In contrast to *TNFSF13B*, the other gene focused on in this study *OAS1*, is rarely defined as a hub gene in SLE or MetS. As the coding gene of 2’-5’-oligoadenylates (2-5As), *OAS1* was proved to be essential for distinctive biological processes, including anti-virus infection, cell growth, as well as cell apoptosis in tumors (36, 37). Recently, a two-sample Mendelian randomization (MR) study revealed that *OAS1* could influence the susceptibility and severity of COVID-19. Another study found a link between *OAS1* genetic risk for Alzheimer’s disease and susceptibility to critical illness with COVID-19 (38, 39). Thomas Magg et al. found that *OAS1* could regulate the development of interferon-induced hyperinflammatory monocytes and B cells (40). These studies indicating that *OAS1* was associated with immune response. In addition, a newly bioinformatics investigation identified *OAS1* as one of the common DEGs with high diagnostic sensitivity and specificity in SLE (AUC > 0.8), which was consistent with our study; nonetheless, it did not reveal the correlation between *OAS1* and immune cell types. Our study ensured the expression of *OAS1* was enriched in monocytes, which was the same as *TNFSF13B*. Also, we found *OAS1* was positively correlated with cholesterol homeostasis and xenobiotic metabolic, suggesting it may be involved in the metabolic disorders in SLE.

It is well known that the Interferon (IFN) signatures are typical features of inflammatory diseases, such as SLE (41). Increased expression of *TNFSF13B* (BAFF), an INF-inducible gene, and activation of the INF pathway are significantly associated with the disease activity of SLE patients (34, 42, 43). *OAS1*, like *TNFSF13B*, also belongs to INF-inducible genes and is responsible for the inflammatory response (38, 39). In MetS, there is growing evidence that the development of MetS is linked to patients’ activated inflammation (44–46). Additionally, recent studies have demonstrated the enhanced level of IFN and the activation of IFN-related pathways in MetS patients, including the cyclic GMP-AMP synthase-stimulator of interferon genes signaling (cGAS-STING) pathways (47, 48). Therefore, it may lead to elevated expression levels of IFN signatures, including *TNFSF13B* and *OAS1*, suggesting that activation of IFN-related pathways may also be an essential feature of MetS, which is the same as SLE.

Cholesterol homeostasis is one of the lipid metabolism pathways. It has been documented that the dysregulation of cholesterol metabolism may be associated with the high incidence of atherosclerosis in SLE patients (49). Moreover, the disorder of cholesterol metabolism may accumulate the burden of inflammation condition, and the dyslipidemia of SLE could also be the result of the immune response in turn (50). Our results showed that cholesterol metabolism was upregulated in SLE and highly enriched in monocytes, which is the same as *TNFSF13B* and *OAS1*. According to previous studies, exogenous chemicals (xenobiotics), including many environmental exposures, consist of one of the risk factors for SLE (51), thus the xenobiotic metabolic dysfunction may also account for the pathogenesis of SLE. We discovered that the metabolism of xenobiotics was markedly increased in SLE, which may be due to the fact that SLE patients were generally exposed to some exogenous poisons and unable to metabolize normally and properly. This study also found that xenobiotic metabolism was positively associated with *TNFSF13B* and *OAS1*. Moreover, xenobiotic metabolism was activated and primarily enriched in monocytes, which was similar to cholesterol metabolism in SLE. Therefore, the use of belimumab and targeted therapy against OAS1 may help restore monocyte metabolic dysfunction and further alleviate the metabolic disorder in SLE patients.

It is worth noting that the data set samples used in our investigation were all drawn from peripheral blood, which has the advantages of being simpler to collect and detect than tissue samples, less hazardous to patients, and having a higher prognostic value. Also, there exist some limitations in our study. Firstly, the sample size of data sets we used was limited, and we were unable to draw a causal association between SLE and MetS due to the absence of a dataset of SLE and MetS comorbidity in public databases. For further validation, larger sample size data sets and comorbidity data sets are needed. Secondly, we lack *in vivo* or *in vitro* experiments to validate our results. Thirdly, the exact mechanisms of metabolic disorders mediated by *TNFSF13B* and *OAS1* and their exact relationship with monocytes need further investigation. Therefore, our results still need to be verified through *in vivo* and *in vitro*.

## Conclusion

In conclusion, this is the first study to screen the shared hub genes and metabolic pathways in peripheral blood for SLE and MetS coexistence. We identified monocyte as the primary cell type, which had a positive correlation with *TNFSF13B* and *OAS1*, as well as cholesterol and xenobiotic metabolism in SLE. This study may provide a new perspective on the pathogenesis and combination treatment of SLE and MetS comorbidity.

## Data availability statement

The datasets presented in this study can be found in online repositories. The names of the repository/repositories and accession number(s) can be found in the article/**Supplementary Material**.

## Author contributions

These authors contributed equally: YW, ZH, YX. YW and XY designed the study and supervised the statistical work. YW, ZH and YX analyzed the data. YW, ZH and YX wrote the manuscript. XY and WW revised and finalized the manuscript. All authors discussed the manuscript.

## Funding

This work was supported by the National Natural Science Foundation of China (81871277). Innovative research team of high-level local universities in Shanghai-Clinical and basic research on the prevention and treatment of some inflammatory diseases by integrative medicine. Huashan Hospital excellent talent training Award Program – “Huajing Award”.

## Acknowledgments

We thank the authors of the GSE72326, GSE98895, GSE81622, and GSE135779 data sets for their contribution.

## Conflict of interest

The authors declare that the research was conducted in the absence of any commercial or financial relationships that could be construed as a potential conflict of interest.

## Publisher’s note

All claims expressed in this article are solely those of the authors and do not necessarily represent those of their affiliated organizations, or those of the publisher, the editors and the reviewers. Any product that may be evaluated in this article, or claim that may be made by its manufacturer, is not guaranteed or endorsed by the publisher.

## Supplementary material

Supplementary Figure 1 Principal components analyses (PCA) performed on GSE72326, GSE98895, and GSE81622. **(A)** PCA before removing batch effects. **(B)** PCA after removing batch effects.

Supplementary Figure 2 ROC curves of 20 shared hub genes.

Supplementary Figure 3 QC of scRNA analysis.

Supplementary Figure 4 UMAP for scRNA-seq data set GSE135779 before cell population annotation, and dot plot of different cell population signatures used for annotation.

Supplementary Table 1 Clinical characteristics of patients and normal controls.
