# Supplementary figures and images for: The shared biomarkers and pathways of systemic lupus erythematosus and metabolic syndrome analyzed by bioinformatics combining machine learning algorithm and single-cell sequencing analysis
Source: Front Immunol. 2022 Oct 19;13:1015882. doi: 10.3389/fimmu.2022.1015882 (PMC9627509; doi:10.3389/fimmu.2022.1015882)

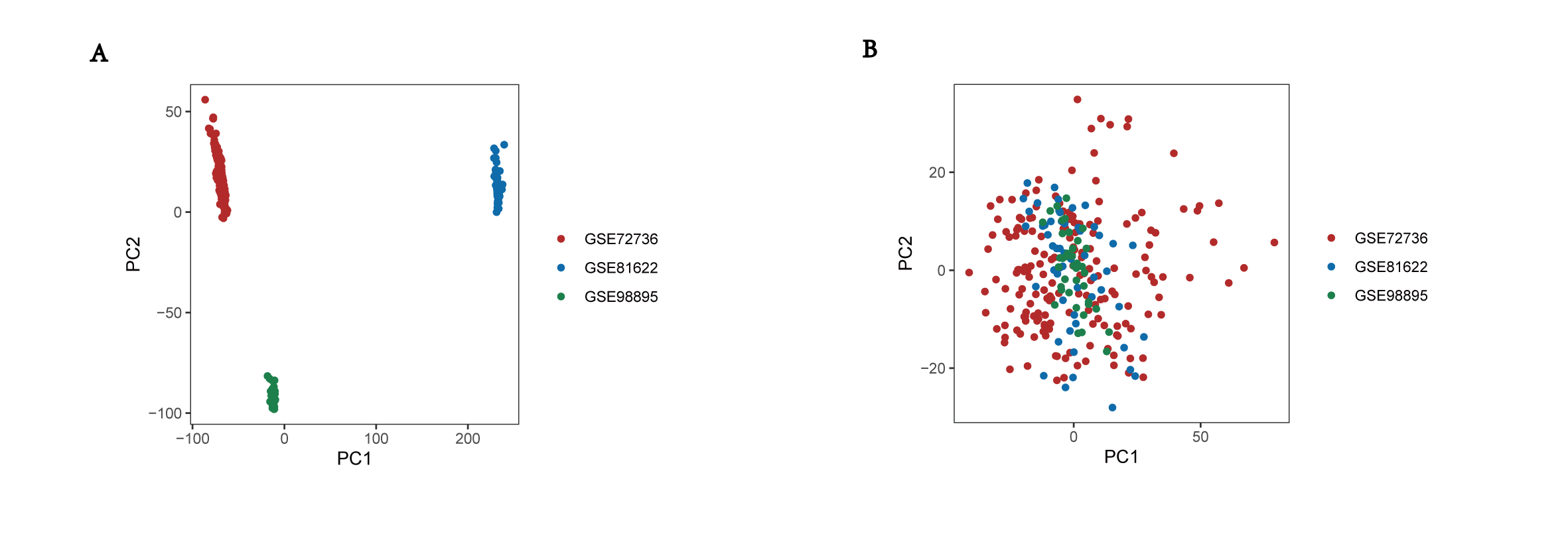

Supplement: Supplementary Figure 1 — Principal components analyses (PCA) performed on GSE72326, GSE98895, and GSE81622. (A) PCA before removing batch effects. (B) PCA after removing batch effects. [file Image_1.tif]

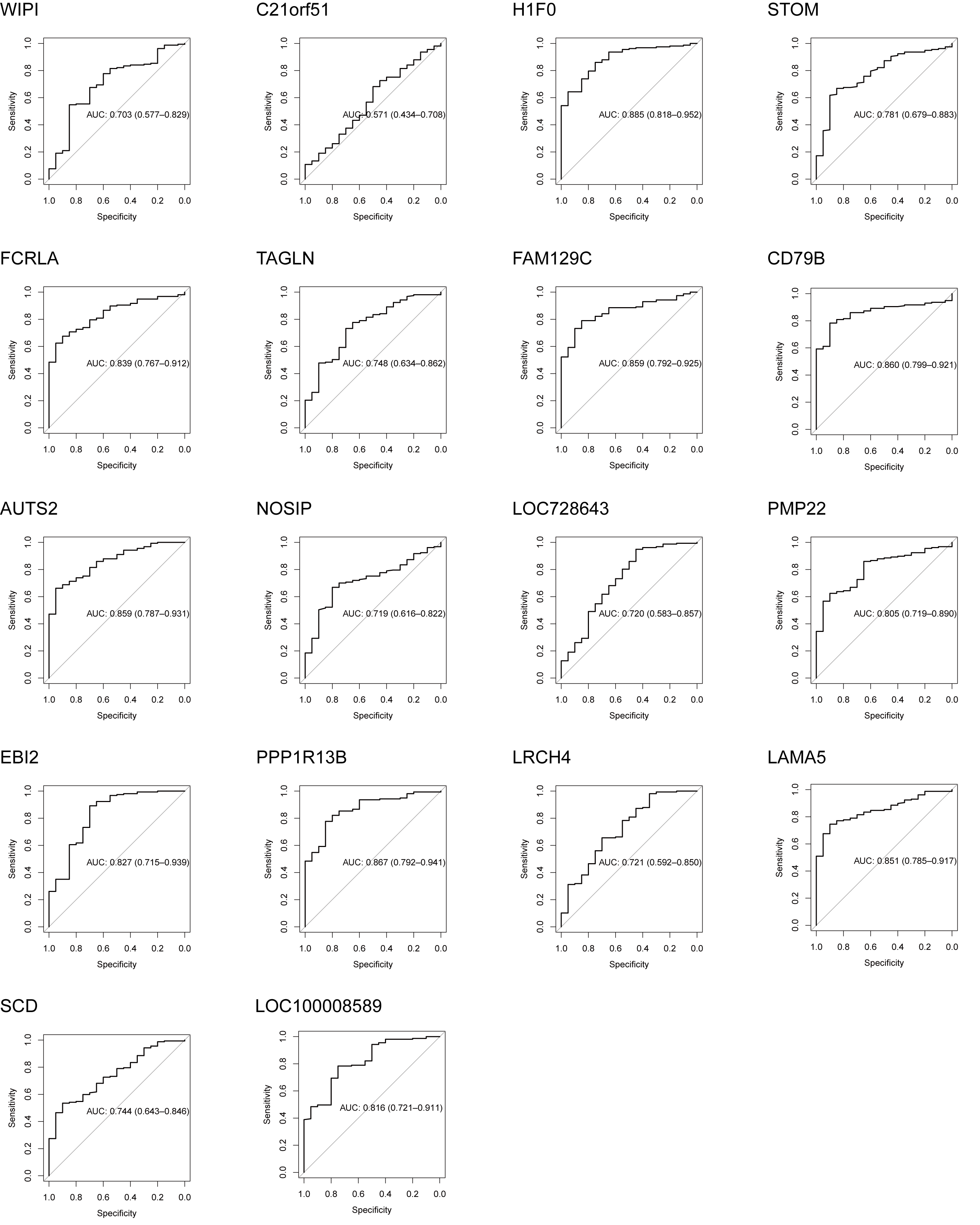

Supplement: Supplementary Figure 2 — ROC curves of 20 shared hub genes. [file Image_2.tif]

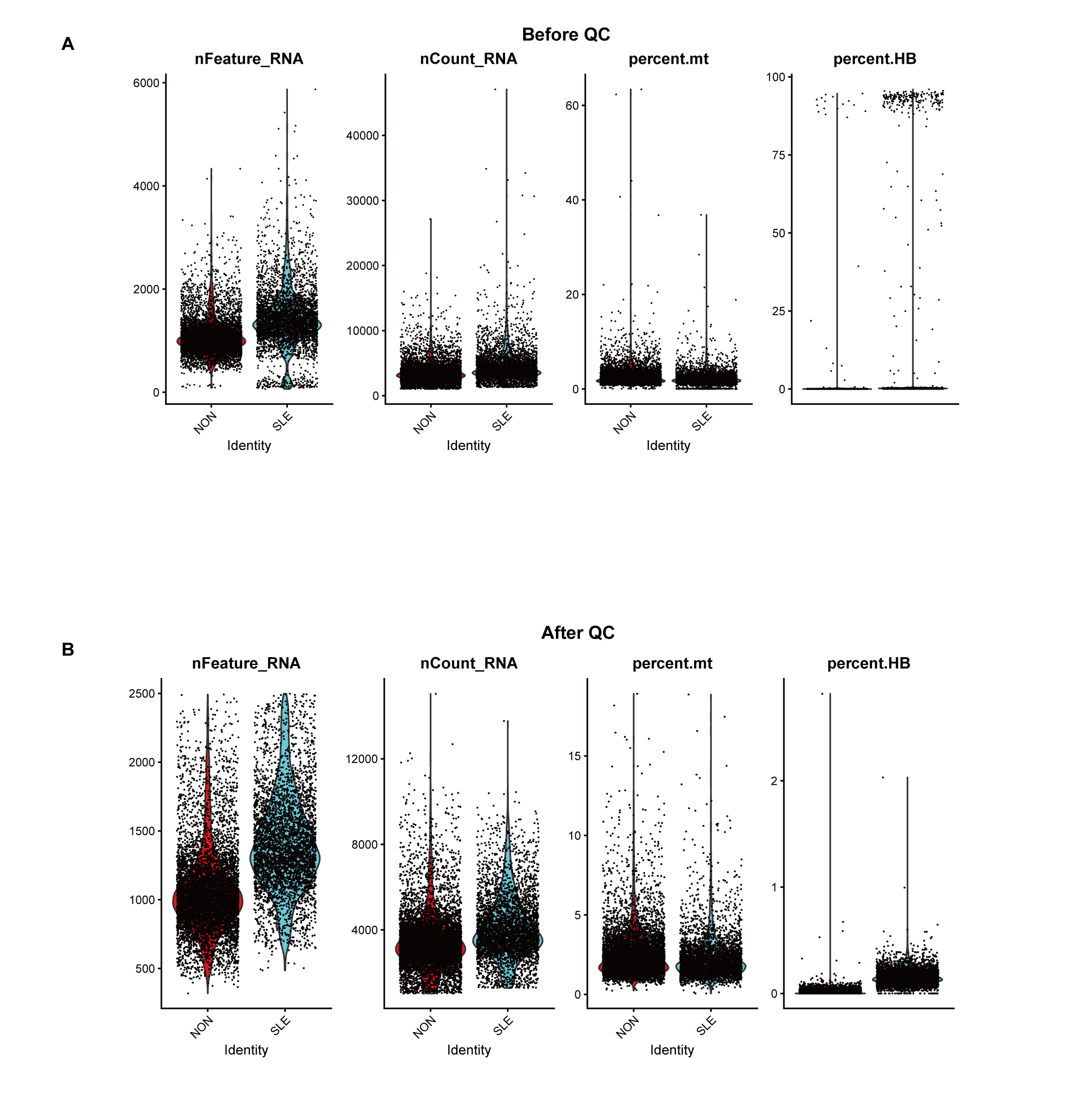

Supplement: Supplementary Figure 3 — QC of scRNA analysis. [file Image_3.tif]

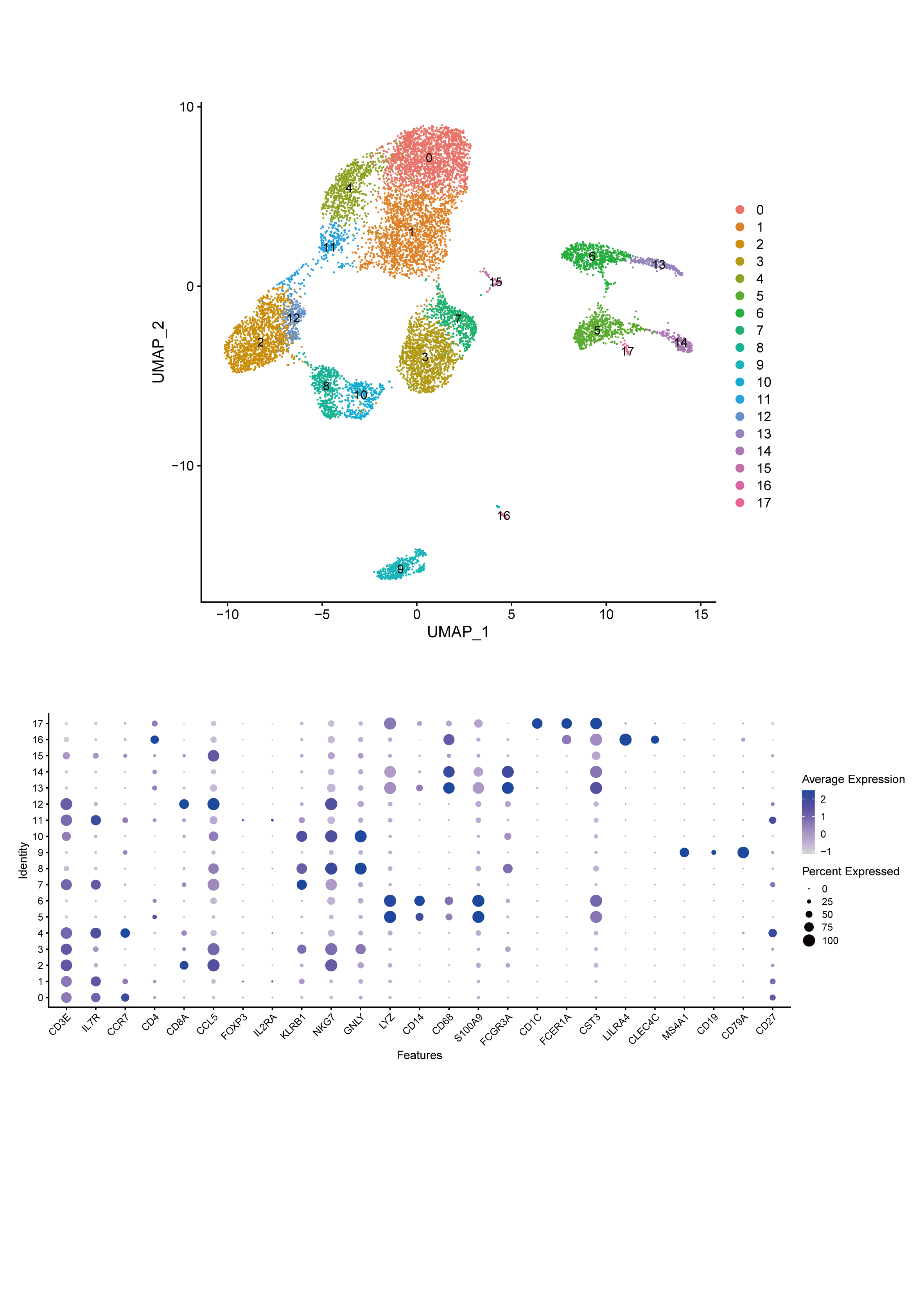

Supplement: Supplementary Figure 4 — UMAP for scRNA-seq data set GSE135779 before cell population annotation, and dot plot of different cell population signatures used for annotation. [file Image_4.tif]

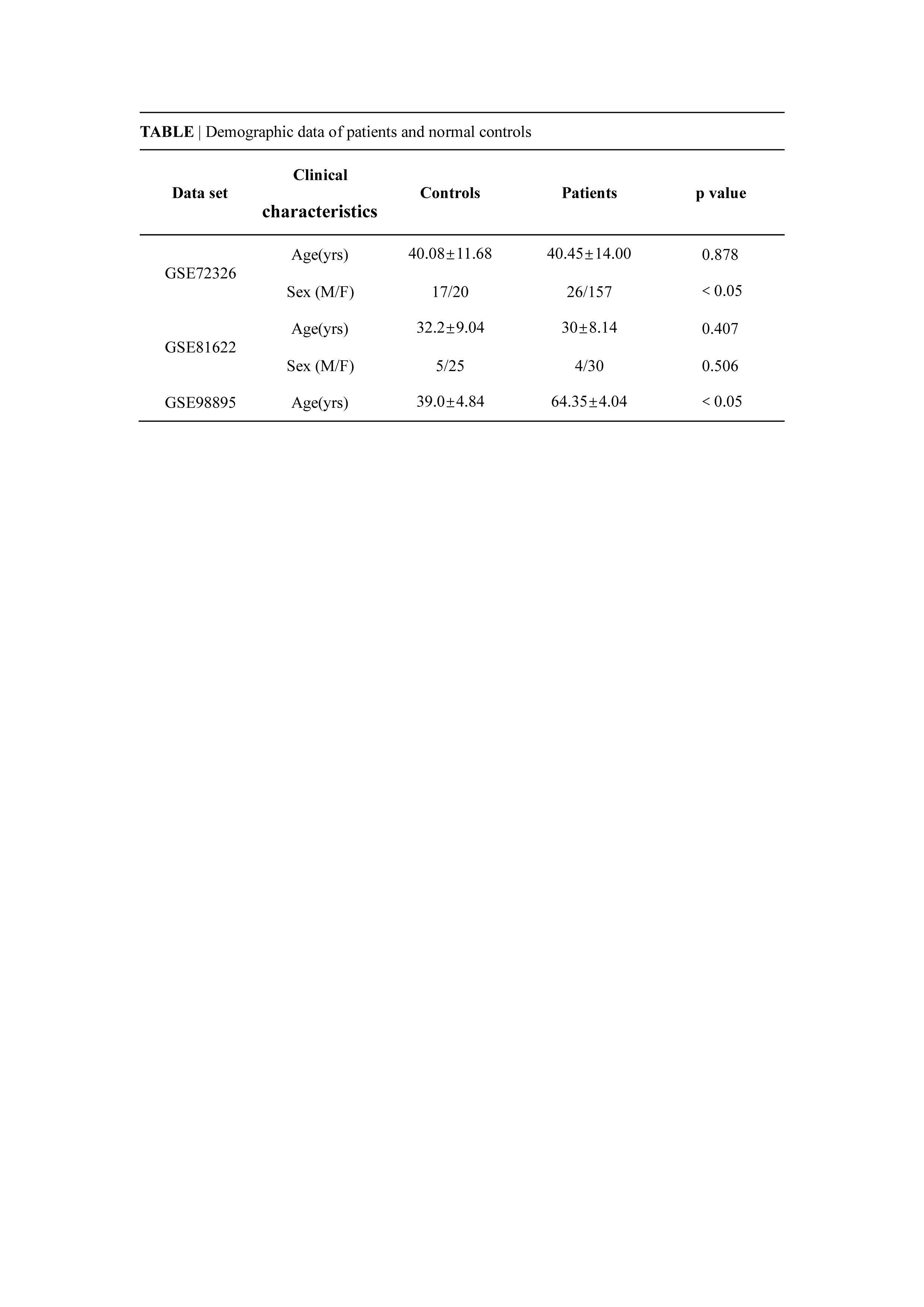

Supplement: Supplementary Table 1 — Clinical characteristics of patients and normal controls. [file Image_5.tif]
